# Third-day weight changes and bronchopulmonary dysplasia risk in preterm infants: a cohort study

**DOI:** 10.3389/fped.2025.1592069

**Published:** 2025-05-27

**Authors:** Wenqian Chen, Wenhong Cai, Zhen Lin, Xiaofeng Ye, Bingjie Chen, Susu Mei, Tingting Huang, Yanli Ren

**Affiliations:** Department of Neonatology, Fujian Maternity and Child Health Hospital, College of Clinical Medicine for Obstetrics & Gynecology and Pediatrics, Fujian Medical University, Fuzhou, Fujian, China

**Keywords:** bronchopulmonary dysplasia, weight change, preterm infants, third-day after birth, predictor

## Abstract

**Objective:**

Fluid balance and weight changes in the early postnatal period are critical indicators of neonatal adaptation and have been implicated in the development of complications in preterm infants. However, the relationship between early weight changes and the risk of bronchopulmonary dysplasia (BPD) remains unclear. This study aimed to evaluate the association between weight change by the third day of life and the subsequent risk of BPD in preterm infants.

**Study Design:**

A retrospective cohort study included preterm infants <32 weeks gestation or <1,500 g birth weight. Logistic regression was used to assess the association between weight change by day 3 (percentage change from birth weight) and BPD.

**Results:**

Among 453 infants, 97.4% (*n* = 441) had weight changes between −15% and 5%, with a BPD incidence of 34.2%. Each 1% increase in weight change by day 3 was linked to a 10% increase in BPD risk (OR = 1.10, 95% CI: 1.03–1.18). Infants without weight loss had a 2.52-fold higher BPD risk (OR = 2.52, 95% CI: 1.34–4.80).

**Conclusion:**

Weight loss byday 3 is associated with a lower BPD risk in preterm infants. The day 3 weight change is a noninvasive and simple early predictor of BPD, and optimizing early fluid management to guide appropriate weight changes may help reduce BPD incidence.

## Introduction

Bronchopulmonary dysplasia (BPD) is a prevalent complication in preterm infants, significantly impacting both survival and long-term health outcomes (1). Globally, the incidence of BPD ranges from 11% to 50% (2). With advancements in perinatal medicine, the survival rate of extremely premature infants has markedly improved, accompanied by a concurrent rise in BPD incidence (3). Pulmonary edema is a hallmark feature of BPD, evident clinically, radiologically, and pathologically (4, 5). This condition is closely linked to early fluid and electrolyte imbalances (6), and strategies such as fluid restriction and diuretic use have been shown to reduce BPD incidence and severity (7–9).

Early weight changes in preterm infants serve as an indicator of fluid balance (6). The initial weight loss, attributed to the contraction of the extracellular compartment and reduction in total body water content, reflects the transition from intrauterine to extrauterine life (10). The degree of weight loss shortly after birth is critical, evidenced by close association with neonatal mortality and the risk of complications. For instance, excessive postnatal weight loss is linked to intraventricular hemorrhage (IVH) (11), whereas insufficient weight loss is associated with persistent ductus arteriosus (PDA) (12). Furthermore, both excessive and inadequate weight loss by day 3 postpartum are correlated with adverse composite outcomes, such as neonatal death or severe neurological impairment (13).

The relationship between early weight changes and BPD remains controversial. For example, William et al. reported that high fluid intake and insufficient weight loss during the first 10 days after birth increase the risk of BPD (6). In contrast, Aksoy et al. found no significant correlation between weight change by day 3 and BPD (14). These conflicting findings highlight the complexity of this issue and underscore the need for further studies to clarify the relationship between early weight change and BPD. Such investigations are essential for developing evidence-based strategies for fluid and electrolyte management in preterm infants.

Preterm infants experience three distinct phases: pre-diuresis, diuresis, and post-diuresis, with the median onset and end of diuresis occurring at approximately 25 and 96 h, respectively (15). During the diuretic phase, urine output remains relatively unresponsive to changes in fluid intake, whether increased or decreased (13, 16). Consequently, improper fluid management during the first three days of life may lead to fluid imbalance.

This study aims to investigate the association between weight changes by day 3 after birth and the risk of BPD in preterm infants, hypothesizing that abnormal weight fluctuations during the first three days postpartum are linked to an increased risk of BPD.

## Materials and methods

### Study design and participants

This retrospective cohort study was conducted at the neonatal intensive care unit (NICU) of Fujian Provincial Maternity and Child Health Hospital (FMCHH) from March 1, 2022, to February 28, 2024. Hospitalization data were retrieved from the electronic medical records system. The NICU at FMCHH is a level III facility, receiving approximately 2,000 preterm infants annually. Participants included preterm infants with a gestational age of less than 32 weeks or a birth weight under 1,500 g who survived beyond 72 h after birth. Exclusion criteria were congenital malformations, genetic metabolic disorders, hospital stays shorter than 28 days despite survival beyond 24 h after discharge, missing data for birth weight, and missing data for weight on day 3 after birth.

### Exposure variables

Body weight was measured using an electronic scale. Birth weight was recorded within one hour of delivery, and the weight on day 3 post-birth was measured at 8:00 AM before feeding. In our NICU, standard feeding times are scheduled at 6:00 AM and 9:00 AM daily, ensuring consistency in weight measurements relative to feeding status. The primary exposure variable was the weight change by day 3, calculated as the percentage difference between day 3 weight and birth weight, normalized to birth weight: Weight change by day 3 (%) = (Day 3 weight−Birth weight)/Birth weight × 100%.

### Primary outcome

The primary outcome was the incidence of bronchopulmonary dysplasia, diagnosed and classified according to the 2001 criteria from the National Institute of Child Health and Human Development (NICHD). BPD was defined as any oxygen dependency (FiO₂ > 21%) at 28 days post-birth. Severity was categorized as mild (no oxygen requirement at 36 weeks corrected gestational age or discharge), moderate (oxygen requirement with FiO₂ < 30%), or severe (oxygen requirement with FiO₂ ≥ 30% or need for positive pressure ventilation) (17). The 2001 definition was adopted because detailed clinical parameters required by the 2018 NICHD criteria (18), such as nasal cannula flow rates and chest imaging findings, were not consistently available in our retrospective dataset. Additionally, a secondary analysis using the 2018 criteria was performed in a subset of infants with complete data to validate the robustness of our findings.

### Secondary outcomes

Secondary outcomes included mortality, length of hospital stay, and the incidences of periventricular leukomalacia (PVL) and necrotizing enterocolitis (NEC) at stage II or higher. Mortality was defined as death during hospitalization or within 24 h after discharge, regardless of postmenstrual age. PVL was diagnosed via cranial ultrasound or magnetic resonance imaging showing periventricular cysts. NEC was staged according to Bell's criteria, with stage II or higher considered significant (19).

### Covariates

Maternal covariates included age, hypertensive disorders of pregnancy (HDP), gestational diabetes mellitus (GDM), antenatal magnesium sulfate, antenatal corticosteroids, chorioamnionitis, prolonged rupture of membranes (PROM) ≥ 18 h, and grade II or higher meconium-stained amniotic fluid. Infant covariates included gestational age (GA), birth weight (BW), sex, small for gestational age (SGA), cesarean section, multiple pregnancy, neonatal asphyxia, postnatal caffeine use, invasive mechanical ventilation (IMV), hemodynamically significant patent ductus arteriosus (hs-PDA), and neonatal respiratory distress syndrome (RDS).

HDP was defined as new-onset hypertension after 20 weeks of gestation (systolic blood pressure ≥140 mmHg and/or diastolic blood pressure ≥90 mmHg) (20). GDM diagnosis followed the IADPSG criteria: fasting blood glucose ≥5.1 mmol/L, 1-hour blood glucose ≥10 mmol/L, or 2-hour blood glucose ≥8.5 mmol/L during a 75-gram oral glucose tolerance test between 24 and 28 weeks gestation (21). Chorioamnionitis was determined using clinical, histopathological, or microbiological evidence (22). PROM ≥ 18 h referred to the interval between spontaneous membrane rupture and labor onset. Antenatal corticosteroids were defined as corticosteroid administration within 7 days before delivery between 24 weeks and 34 weeks gestation. GA was calculated using prenatal ultrasound, menstrual history, or obstetric examination. SGA was defined as birth weight or length below the 10th percentile for gestational age. Neonatal asphyxia was characterized by an Apgar score ≤7 at 1 min post-birth (23). Multiple pregnancy referred to twin or higher-order gestations. IMV was defined as the use of invasive ventilation within 28 days after birth. hs-PDA was diagnosed based on systemic hypoperfusion and echocardiographic criteria, including left atrium-to-aortic root diameter ratio, PDA diameter, and Doppler flow patterns (24). RDS diagnosis was based on clinical presentation, laboratory findings, and chest x-ray results (25). Neonatal sepsis was defined as systemic infection with microbiological evidence (positive cultures from sterile sites such as blood or cerebrospinal fluid) demonstrating pathogenic microorganisms (bacteria, viruses, or fungi) during hospitalization (26).

Management of surfactant, caffeine, and postnatal steroids followed the 2022 European Consensus Guidelines on the Management of Respiratory Distress Syndrome in Preterm Infants (27).

### Statistical methods

To examine the association between weight change by day 3 and BPD incidence, subjects were categorized into seven groups based on weight change rate: <−15%, [−15%, −10%), [−10%, −5%), [−5%, 0%), [0%, 5%), [5%, 10%), and ≥10%. The incidence of BPD was calculated for each group, and a trend graph was generated to preliminarily evaluate the relationship between weight change and BPD risk.

Categorical variables were expressed as frequencies and percentages, with group differences assessed using the *χ^2^ test* or *Fisher's exact test*. Continuous variables were presented as mean ± standard deviation (SD) if normally distributed or as median with interquartile range (IQR) for non-normal distributions. Group comparisons were conducted using *one-way ANOVA* or the *Kruskal–Wallis test*, respectively.

To explore the potential non-linear association between weight change on postnatal day 3 and the risk of bronchopulmonary dysplasia (BPD) in preterm infants, we constructed a restricted cubic spline (RCS) regression model based on logistic regression. The weight change was modeled as a continuous predictor, and BPD was modeled as a binary outcome. Three knots were placed at the 10th, 50th, and 90th percentiles of the weight change rate distribution to allow for flexible modeling of potential non-linear relationships. No covariates were adjusted in the model. The presence of a non-linear association was assessed by performing a likelihood ratio test comparing the model with only the linear term against the model with both linear and spline terms.

If the trend graph suggested a linear relationship between weight change and BPD risk, a generalized linear model (GLM) with logistic regression was employed to estimate regression coefficients. For non-linear relationships, a generalized additive model (GAM) was applied. Subjects were then dichotomized into a “weight loss group” (weight change <0) and a “no weight loss group” (weight change ≥0). Logistic regression was used to compare BPD risk between the groups, calculating odds ratios (OR) and 95% confidence intervals (CI).

Multivariable logistic regression adjusted for potential confounders, including GA, BW, SGA, cesarean section, IMV, PROM ≥18 h, antenatal corticosteroids, antenatal magnesium sulfate, RDS, sepsis, and postnatal caffeine use (28). Subgroup analyses were performed based on GA (<28 weeks or ≥28 weeks), BW (<1,000 g, 1,000–1,500 g, or ≥1,500 g), and SGA status. Interaction tests were conducted to evaluate the relationship between weight change and BPD risk across subgroups.

All analyses were performed using R software (version 4.4.1), and a two-tailed *P*-value <0.05 was considered statistically significant.

## Results

**Figure 1:**
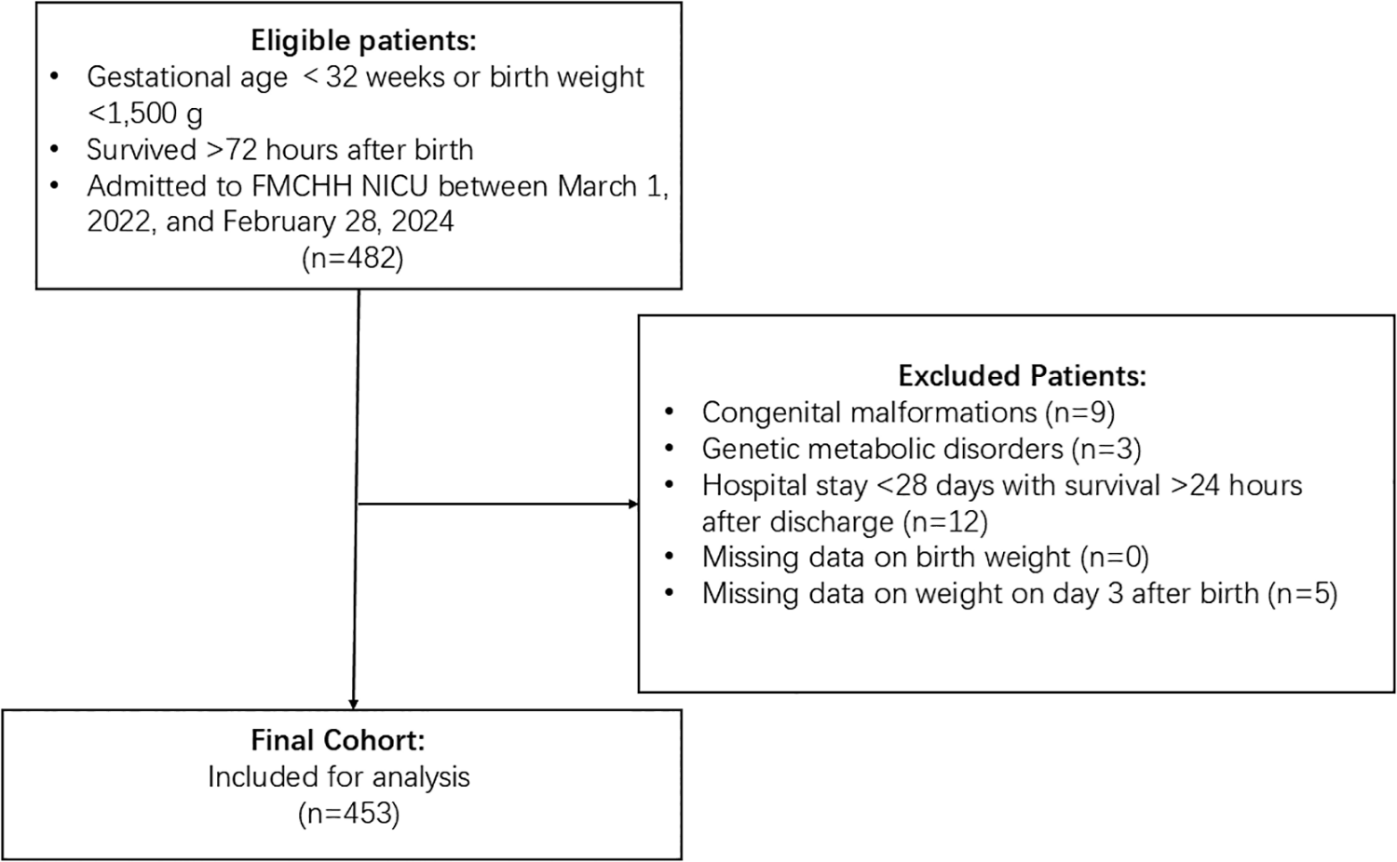
Flow chart of study population.

A total of 482 preterm infants with a gestational age of less than 32 weeks or a birth weight below 1,500 g who survived beyond 72 h after birth were initially enrolled in the study. After excluding 9 cases with congenital malformations, 3 cases with genetic metabolic diseases, 12 cases with hospital stays shorter than 28 days but survival beyond 24 h post-discharge, no cases with missing birth weight data, and 5 cases with missing weight data on the third day of life, 453 cases were included in the final analysis (Figure 1).

**Figure 2:**
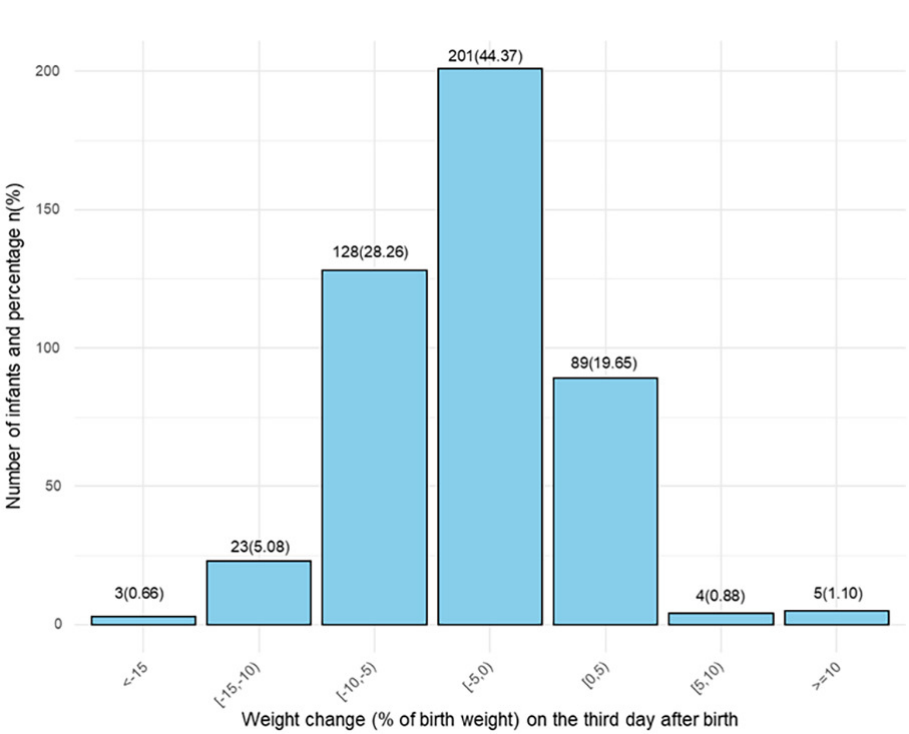
Distribution of weight change by day three of age (expressed as % of birth weight).

The median weight change rate on the third day of life was −3.59% (interquartile range: −5.88%, −0.74%). Among the cohort, 97.4% (*n* = 441) of infants had a weight change rate between −15% and 5%, while 1.99% (*n* = 9) exhibited a weight gain of ≥5%, and only 0.61% (*n* = 3) experienced a weight decrease exceeding 15%. Overall, 78.4% (*n* = 355) of infants experienced weight loss by day 3 of life. The distribution of weight change rates is presented in Figure 2.

Demographic and clinical characteristics of mothers and infants are summarized in Table 1. Maternal prolonged rupture of membranes ≥18 h was more prevalent in infants whose weight change fell within the [−10, 0) range. Infants in this range also showed lower rates of invasive mechanical ventilation (IMV) relative to those outside of this range. Infants with a weight gain of ≥5% were found to have lower gestational ages compared to other groups, and those with smaller birth weights were more likely to experience weight gain. For infants in the [−15, 0) range, lower rates of cesarean delivery and small for gestational age were observed compared to other groups. No significant differences were identified in other maternal and infant characteristics.

**Table 1:** Baseline characteristics of study population categorized by weight change by postnatal day 3.

Variables	Weight change distribution on the third day after birth							
	<−15	[−15, −10)	[−10, −5)	[−5, 0)	[0, 5)	[5, 10)	≥10	*p*
Number of infants *N*	3	23	128	201	89	4	5	
Mother's variables								
mother age [mean (SD)]	27.67 (4.73)	32.17 (4.89)	31.35 (4.68)	31.66 (4.89)	31.75 (4.90)	35.25 (10.08)	32.40 (3.85)	0.535
GDM *n* (%)	2 (66.7)	6 (26.1)	45 (35.2)	69 (34.3)	30 (33.7)	0 (0.0)	1 (20.0)	0.588
HDP *n* (%)	0 (0.0)	4 (17.4)	28 (21.9)	50 (24.9)	19 (21.3)	0 (0.0)	1 (20.0)	0.791
Antenatal corticosteroids use *n* (%)	2 (66.7)	21 (91.3)	106 (82.8)	171 (85.1)	71 (79.8)	4 (100.0)	4 (80.0)	0.705
Magnesium sulfate *n* (%)	2 (66.7)	21 (91.3)	102 (79.7)	151 (75.1)	66 (74.2)	4 (100.0)	4 (80.0)	0.492
PPROM ≥18 h *n* (%)	0 (0.0)	4 (17.4)	43 (33.6)	74 (36.8)	18 (20.2)	2 (50.0)	0 (0.0)	0.023
Amniotic fluid contamination *n* (%)	0 (0.0)	2 (8.7)	4 (3.1)	12 (6.0)	6 (6.8)	1 (25.0)	0 (0.0)	0.472
Chorioamnionitis *n* (%)	1 (33.3)	8 (34.8)	35 (27.6)	59 (29.5)	32 (36.0)	2 (50.0)	0 (0.0)	0.545
Infants’ variables								
GA (weeks) [mean (SD)]	32.05 (1.79)	29.30 (1.68)	29.80 (1.86)	29.56 (2.14)	29.26 (2.30)	26.78 (1.33)	28.46 (1.86)	0.010
BW (gram) [mean (SD)]	1,580.00 (260.00)	1,261.74 (283.61)	1,286.29 (241.64)	1,244.82 (263.39)	1,136.24 (285.83)	780.00 (196.09)	880.00 (180.97)	<0.001
Male *n* (%)	1 (33.3)	8 (34.8)	73 (57.0)	121 (60.2)	58 (65.2)	1 (25.0)	2 (40.0)	0.178
Caesarean *n* (%)	3 (100.0)	13 (56.5)	69 (53.9)	126 (62.7)	61 (68.5)	0 (0.0)	4 (80.0)	0.028
SGA *n* (%)	1 (33.3)	1 (4.3)	13 (10.2)	23 (11.4)	21 (23.6)	0 (0.0)	2 (40.0)	0.014
Multiple pregnancies *n* (%)	2 (66.7)	7 (30.4)	50 (39.1)	60 (29.9)	37 (41.6)	1 (25.0)	2 (40.0)	0.670
Caffeine *n* (%)	2 (66.7)	22 (95.7)	121 (94.5)	175 (87.1)	80 (89.9)	4 (100.0)	5 (100.0)	0.194
Asphyxia *n* (%)	0 (0.0)	2 (8.7)	13 (10.2)	16 (8.0)	10 (11.2)	2 (50.0)	1 (20.0)	0.156
Sepsis *n* (%)	0 (0.0)	2 (8.7)	2 (1.6)	9 (4.5)	2 (2.2)	1 (25)	0 (0.0)	0.128
IMV *n* (%)	0 (0.0)	11 (47.8)	23 (18.0)	53 (26.4)	38 (42.7)	3 (75.0)	2 (40.0)	<0.001
RDS *n* (%)	1 (33.3)	12 (52.2)	50 (39.1)	87 (43.3)	52 (58.4)	3 (75.0)	1 (20.0)	0.066
PDA *n* (%)	0 (0.0)	5 (21.7)	27 (21.1)	51 (25.4)	27 (30.3)	1 (25.0)	2 (40.0)	0.657

**Figure 3:**
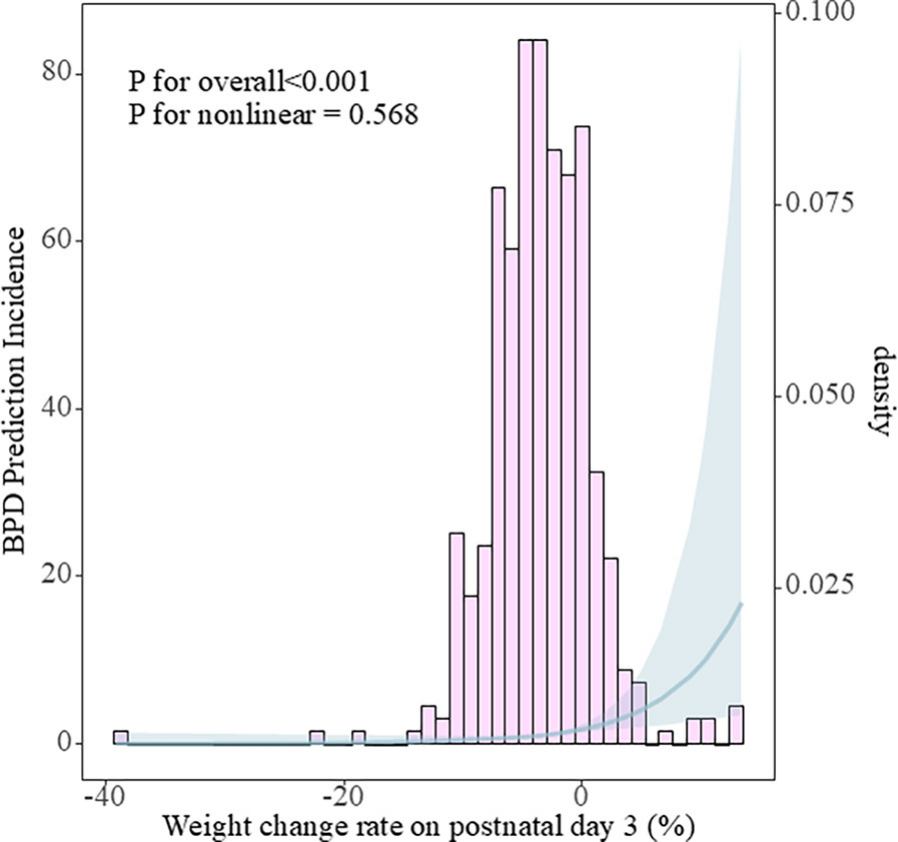
Restricted cubic spline showing the association between weight change rate on postnatal day 3 and the risk of bronchopulmonary dysplasia.

In the restricted cubic spline analysis using three knots, no significant non-linear relationship was identified between weight change on postnatal day 3 and the risk of BPD (*p* for non-linearity = 0.568). The spline curve appeared approximately linear across the range of weight change rates (Figure 3). The overall incidence of bronchopulmonary dysplasia was 155/453 (34.2%). As the weight change increased, the incidence of BPD also rose, displaying an approximately linear relationship within a certain range (Supplementary Figure S1). Infants who experienced weight loss by the third day had significantly shorter hospital stays compared to those who gained weight (Table 2). A generalized linear model (GLM) with logistic regression indicated that for every 1% increase in weight change rate, the risk of BPD increased by 11% (OR 1.16, 95% CI 1.09–1.22). After adjusting for confounders—including gestational age (GA), birth weight (BW), SGA, cesarean section, IMV, PROM ≥18 h, antenatal corticosteroid administration, antenatal magnesium sulfate administration, respiratory distress syndrome (RDS), sepsis, and caffeine use—the association remained significant. The risk of moderate to severe BPD increased by 11% (OR 1.11, 95% CI 1.03–1.19), and the risk of severe BPD increased by 20% (OR 1.20, 95% CI 1.09–1.32) per 1% increase in weight change rate. A secondary analysis applying the 2018 NICHD BPD criteria was conducted in a subset of infants (*n* = 181) with complete relevant data. The association between weight change and BPD risk remained consistent in this subgroup. Detailed results are presented in Supplementary Table S1–3.

**Table 2:** Univariate analysis of primary and secondary outcomes.

Outcomes		Weight change distribution on the third day after birth							
		<−15	[−15, −10)	[−10, −5)	[−5, 0)	[0, 5)	[5, 10)	≥10	*p*
Number of infants *N*		3	23	128	201	89	4	5	
Primary outcome									
BPD *n* (%)		0 (0.0)	4 (17.4)	37 (28.9)	59 (29.4)	48 (53.9)	3 (75.0)	4 (80.0)	<0.001
BPD_grade *n* (%)	Mild	0 (0.0)	1 (4.3)	22 (17.2)	41 (20.4)	20 (22.5)	0 (0.0)	0 (0.0)	<0.001
	Moderate	0 (0.0)	1 (4.3)	10 (7.8)	12 (6.0)	9 (10.1)	0 (0.0)	1 (20.0)	<0.001
	Severe	0 (0.0)	2 (8.7)	5 (3.9)	6 (3.0)	19 (21.3)	3 (75.0)	3 (60.0)	<0.001
Secondary outcomes									
NEC *n* (%)		0 (0.0)	8 (34.8)	30 (23.4)	37 (18.4)	14 (15.7)	2 (50.0)	1 (20.0)	0.217
PVL *n* (%)		0 (0.0)	1 (4.3)	0 (0.0)	4 (2.0)	1 (1.1)	0 (0.0)	0 (0.0)	0.65
Dead *n* (%)		0 (0.0)	0 (0.0)	0 (0.0)	1 (0.5)	1 (1.2)	0 (0.0)	0 (0.0)	1.94
Length_stay (days) [mean (SD)]		56.00 (12.29)	55.17 (15.74)	53.36 (21.65)	54.89 (21.27)	62.81 (26.61)	91.75 (24.32)	79.00 (20.92)	<0.001

Linear regression analysis also revealed that higher weight changes were associated with longer hospital stays, but this relationship lost significance after adjusting for confounders (*β* 0.97, 95% CI 0.71–1.31). Other clinical outcomes, including necrotizing enterocolitis (NEC), periventricular leukomalacia (PVL), and mortality, showed no significant differences across weight change rate groups (Table 3).

**Table 3:** Multivariate analysis of primary and secondary outcomes.

Outcomes	Crude OR (95% Cl)	Adjust OR (95% Cl)^a^	Adjust OR (95% Cl)^b^
BPD	1.16 (1.09, 1.22)	1.10 (1.02, 1.17)	1.10 (1.03, 1.18)
BPD moderate to severe	1.19 (1.11, 1.27)	1.11 (1.03, 1.19)	1.11 (1.03, 1.19)
BPD severe	1.25 (1.15, 1.36)	1.20 (1.09, 1.32)	1.20 (1.09, 1.32)
Secondary outcomes			
NEC	0.98 (0.93, 1.03)	0.96 (0.91, 1.01)	0.96 (0.91, 1.01)
PVL	1.03 (0.85, 1.24)	1.05 (0.85, 1.30)	1.05 (0.85, 1.30)
Dead	1.15 (0.88, 1.51)	1.12 (0.72, 1.72)	1.12 (0.72, 1.72)
Length_stay	2.73 (1.72, 4.34)^b^	0.97 (0.71, 1.31)^c^	0.97 (0.71, 1.31)^c^

^a^ Adjust for GA, BW, SGA, PPROM, IMV, RDS, steroid hormone, magnesium sulfate, caffeine and caesarean.

^b^ Adjust for GA, BW, SGA, PPROM, IMV, RDS, sepsis, steroid hormone, magnesium sulfate, caffeine and caesarean.

^c^ Linear regression.

Further analysis categorized infants into two groups based on whether their weight on the third day was lower than their birth weight: the “weight loss group” and the “no weight loss group.” Compared to the weight loss group, the no weight loss group had higher rates of maternal PROM ≥18 h, lower GA and BW, higher SGA incidence, increased IMV rates, and a higher prevalence of RDS. The no weight loss group also demonstrated a significantly higher incidence of BPD (Table 4).

**Table 4:** Baseline characteristics and outcomes stratified by postnatal day 3 weight change status (weight loss vs. no weight loss relative to birth weight).

Variables	Weight loss on the third day after birth (*N* = 355)	Weight did not decrease on the third day after birth (*N* = 98)	*p*
Mother's variables			
Mother age [mean (SD)]	31.55 (4.81)	31.93 (5.10)	0.493
GDM *n* (%)	122 (34.4)	31 (31.6)	0.7
HDP *n* (%)	82 (23.1)	20 (20.4)	0.669
Antenatal corticosteroids use *n* (%)	300 (84.5)	79 (80.6)	0.442
Magnesium sulfate *n* (%)	276 (77.7)	74 (75.5)	0.74
PPROM ≥19 h *n* (%)	121 (34.1)	20 (20.4)	0.014
Amniotic fluid Contamination *n* (%)	18 (5.1)	7 (7.2)	0.569
Chorioamnionitis (%)	103 (29.2)	34 (34.7)	0.354
Infants’ variables			
GA [mean (SD)]	29.65 (2.02)	29.11 (2.29)	0.024
BW [mean (SD)]	1,263.70 (258.37)	1,108.62 (290.70)	<0.001
Male *n* (%)	203 (57.2)	62 (63.3)	0.334
Caesarean *n* (%)	211 (59.4)	65 (66.3)	0.262
SGA *n* (%)	38 (10.7)	23 (23.5)	0.002
Multiple pregnancies *n* (%)	119 (33.5	40 (40.8)	0.316
Caffeine *n* (%)	320 (90.1)	89 (90.8)	0.994
Asphyxia *n* (%)	31 (8.7)	13 (13.3)	0.251
Sepsis *n* (%)	13 (3.7)	3 (3.1)	1.000
IMV *n* (%)	87 (24.5)	43 (43.9)	<0.001
RDS *n* (%)	150 (42.3)	56 (57.1)	0.012
PDA *n* (%)	83 (23.4)	30 (30.6)	0.183
Outcomes			
BPD *n* (%)	100 (28.2)	55 (56.1)	<0.002
BPD_grade *n* (%)			
Mild	64 (18.0)	20 (20.4)	<0.001
Moderate	23 (6.5)	10 (10.2)	<0.001
Severe	13 (3.7)	25 (25.5)	<0.001
NEC *n* (%)	75 (21.1)	16 (16.3)	0.364
PVL *n* (%)	5 (1.4)	1 (1.0)	1
Dead *n* (%)	1 (0.3)	1 (1.1)	0.889
Length_stay [mean (SD)]	54.37 (20.99)	64.82 (26.88)	<0.001

**Figure 4:**
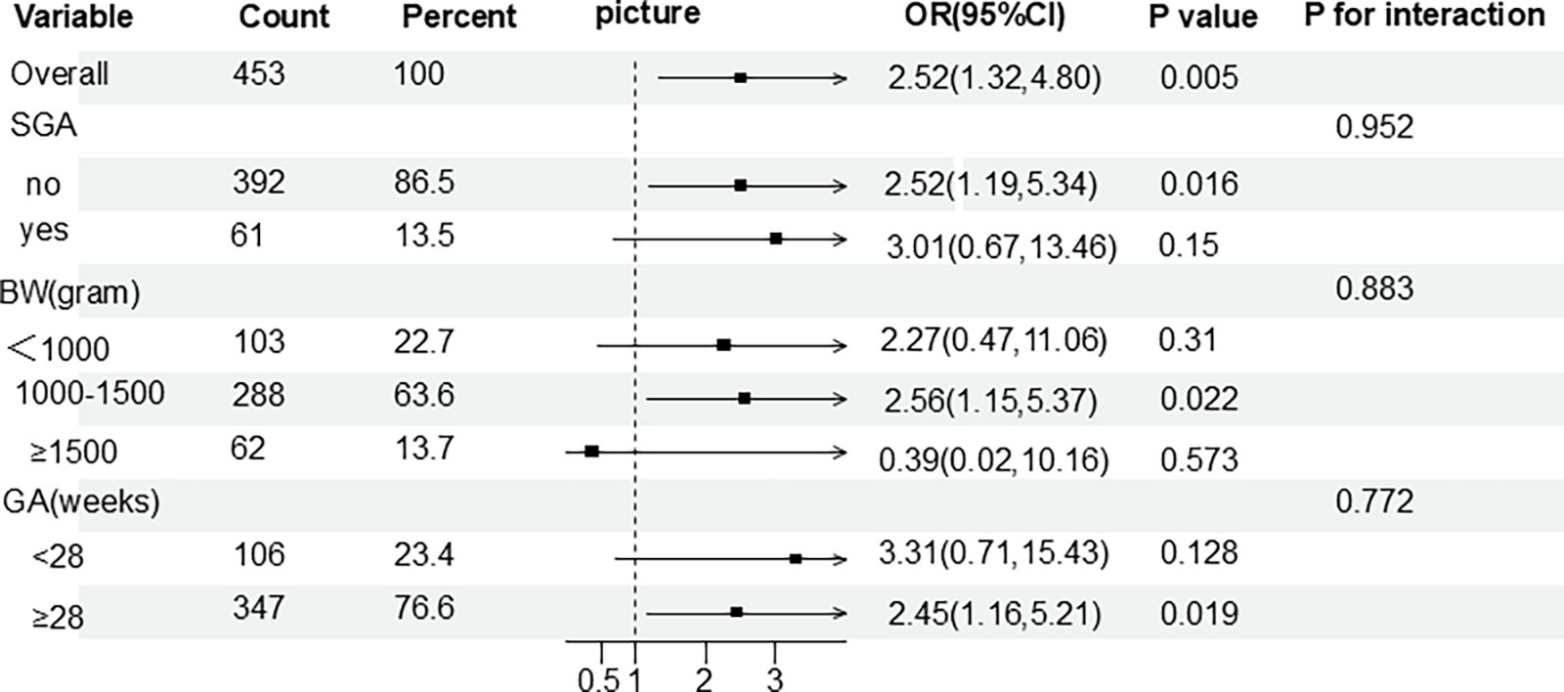
Subgroup analysis forest plot of BPD risk by postnatal day 3 weight change rate (weight loss vs. no weight loss relative to birth weight). SGA, small for gestational age; BW, birth weight; GA, gestational age.

Subgroup analysis stratified by GA (<28 weeks or ≥28 weeks), BW (<1,000 g, 1,000–1,500 g, or ≥1,500 g), and SGA status showed that no weight loss was a risk factor for BPD across most subgroups. Specifically, in both the GA <28 weeks and GA ≥28 weeks subgroups, no weight loss was associated with an increased risk of BPD. Similarly, in the BW <1,000 g and 1,000–1,500 g subgroups, no weight loss was a risk factor for BPD. However, in the BW ≥1,500 g subgroup, no weight loss appeared to have a protective effect, though this finding was not statistically significant. Both SGA and non-SGA subgroups demonstrated a higher risk of BPD associated with no weight loss. Interaction tests revealed no significant interaction between GA, BW, or SGA and the presence or absence of weight loss on the third day of life (Figure 4).

## Discussion

Our study found that less weight loss or weight gain on the third day after birth (relative to birth weight) is associated with a higher risk of BPD. After adjusting for confounding factors such as GA, BW, SGA, cesarean delivery, IMV, maternal PROM ≥18 h, prenatal corticosteroids and magnesium sulfate, RDS, sepsis, and postnatal caffeine, we observed that for every 1% decrease weight loss or 1% weight gain by day 3, the risk increased by 1.10-fold for total BPD, 1.11-fold for moderate-to-severe BPD, and 1.20-fold for severe BPD.Additionally, infants with no weight loss by day 3 had a 2.52 times higher risk of BPD compared to those who experienced weight loss. However, no significant correlation was found between weight change by day 3 and the incidence of NEC, PVL, mortality, or length of hospitalization.

Previous studies examining the relationship between early postnatal weight change and BPD have produced mixed results. Verma et al. suggested that weight loss in the early postnatal period (6–10 days) reduced the risk of BPD (29). William et al. (6) assessed the relationship between fluid intake, weight loss, and BPD risk in the first 10 days and found that higher fluid intake and less weight loss were associated with increased BPD risk. A secondary analysis of a European multi-center randomized clinical trial also indicated that weight gain by day 3 was linked to a higher need for mechanical ventilation and increased BPD risk by day 14 in extremely preterm infants (30). These findings align with our results. However, other studies have not found a significant association between weight change and BPD risk. For example, a case-control study did not find early postnatal weight change to be a risk factor for BPD (31), possibly due to selection bias inherent in case-control designs. Additionally, Aksoy et al. (14) found no significant relationship between weight change by day 3 and BPD in a study of 129 extremely low birth weight (ELBW) infants. They defined abnormal weight change as <3% or >12% weight loss (compared to a normal range of 3%–12%) but observed no significant difference in BPD incidence between groups (9 vs. 14 cases). However, their analysis may have been underpowered due to the small sample size and low BPD incidence. Additionally, their stratification criteria differed fundamentally from the design of our study. Similarly, Zozaya et al. (13) found no significant association between weight change and BPD in preterm infants <29 weeks' gestation. Their primary focus was on the association between day 3 weight change and mortality and/or severe neurological injury, with BPD analyzed as a secondary outcome. Limited adjustment for confounders such as mechanical ventilation and differences in study populations may have contributed to the lack of association. Further research with larger samples and more rigorous designs is warranted to better elucidate the relationship between early postnatal weight dynamics and BPD risk. SGA infants typically have lower lean mass and fat content, with higher water content compared to appropriate for gestational age (AGA) infants (32, 33). They usually experience less weight loss than AGA infants (34). Wadhawan et al. (35) found that in extremely preterm infants, weight loss in the first 10 days was associated with lower mortality or BPD risk, regardless of SGA or AGA status. Our subgroup analysis confirmed that the proportion of SGA infants was higher in the no-weight-loss group, and weight loss by day 3 was a protective factor for BPD in both SGA and AGA subgroups. Previous studies have also suggested that early weight loss in ELBW infants (BW < 1,000 g) is associated with a lower BPD risk (14, 29). In our study, weight loss by day 3 was found to be a protective factor for BPD in infants with birth weights <1,000 g and between 1,000–1,500 g, though no significant difference was found in the ≥1,500 g subgroup. The small sample size in this subgroup may have contributed to the lack of statistical significance, warranting further investigation. The relationship between early postnatal weight change and BPD may be influenced by fluid imbalance. Previous studies have suggested that less weight loss or weight gain by day 3 may reflect a positive fluid balance (36). Positive fluid balance in early life is associated with an increased incidence of BPD (37, 38). Excess fluid may lead to pulmonary edema and reduced lung compliance, which increases the need for ventilatory support and oxygen, thereby contributing to lung injury and BPD development (35).Previous research has identified a U-shaped relationship between early postnatal weight change and mortality or severe neurological injury (13), but few studies have specifically examined the relationship between weight change and PVL. Our study did not find a significant association between weight change by day 3 and PVL or mortality, possibly due to the low incidence of these outcomes in our cohort and insufficient statistical power. This warrants further research in larger populations.

In our study, we found a positive correlation between weight gain by day 3 and length of hospitalization in univariate logistic regression. However, this association was not significant after adjusting for confounders, suggesting that factors such as gestational age, birth weight, postnatal nutrition, and complications in preterm infants may have a greater impact on hospitalization duration. Research on the relationship between NEC and weight change is limited. Bell et al. (39) found that excessive fluid intake on day 3 was associated with higher NEC rates, while Lorenz et al. (40) found no correlation between weight loss and NEC in the first five days, which is consistent with our findings. Nonetheless, our study has several limitations. Typically, full-term infants should not lose more than 10% of their birth weight in the first week post-birth, while for extremely preterm infants, a weight loss of 10%–20% may be beneficial (36). In our study, only 2.6% of the cohort had weight loss exceeding 15% or weight gain exceeding 10%, so the relationship between weight change in these ranges and BPD could not be clearly defined. However, previous studies have not found a U-shaped non-linear relationship between weight change by day 3 and BPD (13). Our results suggest that in the range of [−15%, 10%), an increased weight loss percentage by postnatal day 3 is associated with a decreased risk of BPD.

BPD development is influenced by multiple factors, including early fluid intake (6, 41), feeding practices (42), nutritional support (43), and maternal health and nutrition during pregnancy (44, 45). The absence of detailed records on these variables in our retrospective cohort may have introduced residual confounding. Intrauterine tobacco exposure can impair fetal growth and neonatal respiratory function, significantly increasing BPD risk (46, 47). Although we lacked data on maternal smoking, the relatively low smoking prevalence among Chinese women may have minimized its impact (48, 49).Neonatal sepsis affects weight trajectories via systemic inflammation and catabolic stress, contributing to lung injury and BPD (45, 50). Although we adjusted for sepsis, limited timing data may have led to inclusion of late-onset cases, affecting accuracy. Antibiotic exposure can alter metabolism and disrupt the gut microbiota, increasing BPD risk (44, 51). Due to incomplete data on antibiotic use, this potential confounder was not controlled. Future prospective studies should address these limitations by systematically collecting and adjusting for relevant perinatal variables.

Our study was conducted in a single-center NICU, which may limit generalizability and introduce selection bias, as referred preterm infants often present with more severe conditions. While our center is a level III regional referral hospital admitting ∼2,000 preterm infants annually and thus offers a broadly representative cohort, future multicenter trials involving more diverse populations are needed to confirm and expand upon our findings.

Despite its limitations, our study highlights the clinical relevance of early postnatal weight dynamics in predicting BPD risk. Monitoring weight change by day 3 offers a simple, non-invasive tool for early risk stratification, potentially guiding individualized fluid management and timely interventions to mitigate BPD (6).

While we identified a significant association between day 3 weight change and BPD, evaluating weight trends over multiple time points—such as across the first week—may better capture fluid balance dynamics and enhance predictive accuracy (52). Future studies should incorporate serial weight monitoring to refine this association.

Moreover, early postnatal weight changes may have broader implications beyond BPD, potentially influencing long-term lung function (53), neurodevelopmental (54), and growth outcomes (55). However, our retrospective design and limited follow-up precluded assessment of these outcomes. Future large-scale, prospective studies with extended follow-up are needed to explore the long-term impact of early weight dynamics.

## Conclusion

Our study found that weight change on the third day after birth in preterm infants is closely associated with the risk of BPD, with insufficient weight loss potentially increasing the risk of BPD. Dynamic monitoring of weight change and optimizing fluid management strategies may help reduce the incidence of BPD.

## Data Availability

The raw data supporting the conclusions of this article will be made available by the authors, without undue reservation.

## Supplementary material

Supplementary Figure S1 Incidence of BPD by postnatal day 3 weight change categories.
