# Supplementary material for: Third-day weight changes and bronchopulmonary dysplasia risk in preterm infants: a cohort study
Source: Front Pediatr. 2025 May 27;13:1592069. doi: 10.3389/fped.2025.1592069 (PMC12148873; doi:10.3389/fped.2025.1592069)
Supplement: Supplementary file 1 [file Table1.docx]

Supplementary table 1 Baseline characteristics of the population diagnosed with 2018 NICHD BPD criteria.

| Variables | Weight change distribution on the third day after birth | | | | | | | |
| --- | --- | --- | --- | --- | --- | --- | --- | --- |
|  | <-15 | [-15,-10) | [-10,-5) | [-5,0) | [0,5) | [5,10) | >=10 | p |
| Number of Infants N | 2 | 11 | 49 | 73 | 40 | 3 | 3 |  |
| Mother’s variables |  |  |  |  |  |  |  |  |
| mother age (mean (SD)) | 29.50 (4.95) | 30.64 (5.57) | 31.10 (5.18) | 32.33 (4.57) | 32.20 (4.47) | 30.67 (5.13) | 32.67 (4.51) | 0.733 |
| GDM n(%) | 1 ( 50.0) | 3 ( 27.3) | 16 ( 32.7) | 30 ( 41.1) | 15 ( 37.5) | 0 ( 0.0) | 1 ( 33.3) | 0.771 |
| HDP n(%) | 0 ( 0.0) | 1 ( 9.1) | 13 ( 26.5) | 19 ( 26.0) | 12 ( 30.0) | 0 ( 0.0) | 1 ( 33.3) | 0.698 |
| Antenatal corticosteroids use n(%) | 2 (100.0) | 11 (100.0) | 41 ( 83.7) | 62 ( 84.9) | 30 ( 75.0) | 3 (100.0) | 2 ( 66.7) | 0.427 |
| Magnesium sulfate n(%) | 2 (100.0) | 10 ( 90.9) | 36 ( 73.5) | 52 ( 71.2) | 31 ( 77.5) | 3 (100.0) | 3 (100.0) | 0.551 |
| PPROM ≥18 hours n(%) | 0 ( 0.0) | 3 ( 27.3) | 15 ( 30.6) | 23 ( 31.5) | 6 ( 15.0) | 1 ( 33.3) | 0 ( 0.0) | 0.428 |
| amniotic fluid contamination n(%) | 0 ( 0.0) | 0 ( 0.0) | 1 ( 2.0) | 4 ( 5.5) | 2 ( 5.0) | 0 ( 0.0) | 0 ( 0.0) | 0.933 |
| chorioamnionitis n(%) | 1 ( 50.0) | 3 ( 27.3) | 13 ( 26.5) | 21 ( 28.8) | 13 ( 32.5) | 2 ( 66.7) | 0 ( 0.0) | 0.664 |
| Infants’ variables |  |  |  |  |  |  |  |  |
| GA (weeks) (mean (SD)) | 32.08 (2.52) | 29.38 (1.71) | 29.86 (1.88) | 29.57 (2.22) | 29.39 (2.38) | 27.00 (1.54) | 28.43 (2.51) | 0.170 |
| BW (gram) (mean (SD)) | 1450.00 (183.85) | 1223.64 (263.15) | 1285.61 (249.11) | 1294.32 (245.13) | 1156.88 (277.98) | 783.33 (240.02) | 993.33 (130.13) | 0.001 |
| male n(%) | 1 ( 50.0) | 4 ( 36.4) | 28 ( 57.1) | 53 ( 72.6) | 19 ( 47.5) | 2 ( 66.7) | 2 ( 66.7) | 0.110 |
| Caesarean n(%) | 2 (100.0) | 7 ( 63.6) | 28 ( 57.1) | 43 ( 58.9) | 29 ( 72.5) | 0 ( 0.0) | 3 (100.0) | 0.101 |
| SGA n(%) | 0 ( 0.0) | 1 ( 9.1) | 7 ( 14.3) | 6 ( 8.2) | 9 ( 22.5) | 0 ( 0.0) | 1 ( 33.3) | 0.359 |
| Multiple pregnancies n(%) | 1 ( 50.0) | 5 ( 45.5) | 18 ( 36.7) | 23 ( 31.5) | 17 ( 42.5) | 1 ( 33.3) | 1 ( 33.3) | 0.922 |
| Caffeine n(%) | 2 (100.0) | 11 (100.0) | 45 ( 91.8) | 63 ( 86.3) | 36 ( 90.0) | 3 (100.0) | 3 (100.0) | 0.755 |
| Asphyxia n(%) | 0 ( 0.0) | 1 ( 9.1) | 4 ( 8.2) | 8 ( 11.0) | 4 ( 10.0) | 2 ( 66.7) | 0 ( 0.0) | 0.089 |
| Sepsis n(%) | 0 ( 0.0) | 2 ( 18.2) | 1 ( 2.0) | 3 ( 4.1) | 1 ( 2.5) | 1 ( 33.3) | 0 ( 0.0) | 0.059 |
| IMV n(%) | 0 ( 0.0) | 7 ( 63.6) | 10 ( 20.4) | 16 ( 21.9) | 16 ( 40.0) | 2 ( 66.7) | 2 ( 66.7) | 0.009 |
| RDS n(%) | 1 ( 50.0) | 5 ( 45.5) | 18 ( 36.7) | 29 ( 39.7) | 29 ( 72.5) | 2 ( 66.7) | 1 ( 33.3) | 0.022 |
| PDA n(%) | 0 ( 0.0) | 3 ( 27.3) | 10 ( 20.4) | 20 ( 27.4) | 13 ( 32.5) | 0 ( 0.0) | 1 ( 33.3) | 0.730 |

Supplementary table 2. Univariate analysis of primary outcome (2018 NICHD BPD criteria).

| Outcomes | | Weight change distribution on the third day after birth | | | | | | | | |
| --- | --- | --- | --- | --- | --- | --- | --- | --- | --- | --- |
|  |  | <-15 | [-15,-10) | [-10,-5) | [-5,0) | [0,5) | [5,10) | >=10 | | p |
| Number of Infants N | | 2 | 11 | 49 | 73 | 40 | 3 | 3 | |  |
| Primary outcome |  |  |  |  |  |  |  | |  |  |
| BPD n(%) |  | 0 (0.0) | 2 (18.2) | 10 (20.4) | 20 (27.4) | 22 (55.0) | 2 (66.7) | 2 (66.7) | | 0.005 |
| BPD_grade n(%) | Mild | 0 (0.0) | 0 (0.0) | 5 (10.2) | 19 (26.0) | 8 (20.0) | 0 (0.0) | 0 (0.0) | | <0.001 |
|  | Moderate | 0 (0.0) | 1 (9.1) | 5 (10.2) | 0 (0.0) | 3 (7.5) | 0 (0.0) | 0 (0.0) | | <0.001 |
|  | Severe | 0 (0.0) | 1 (9.1) | 0 (0.0) | 1 (1.4) | 11 (27.5) | 2 (66.7) | 2 (66.7) | | <0.001 |

Supplementary table 3 Multivariate analysis of primary outcome (2018 NICHD BPD criteria).

| Outcomes | crude OR (95%Cl) | adjust OR (95%Cl)^1^ | adjust OR (95%Cl)^2^ |
| --- | --- | --- | --- |
| BPD | 1.17 (1.08, 1.27) | 1.14 (1.03, 1.26) | 1.15 (1.03, 1.27) |
| BPD moderate to severe | 1.19 (1.08, 1.32) | 1.14 (1.02, 1.28) | 1.14 (1.02, 1.28) |
| BPD severe | 1.33 (1.16, 1.53) | 1.30 (1.09, 1.55) | 1.32 (1.10, 1.58) |

^1^ adjust for GA,BW,SGA,PPROM,IMV,RDS,steroid hormone, magnesium sulfate, caffeine and caesarean.

^2^ adjust for GA,BW,SGA,PPROM,IMV,RDS,sepsis,steroid hormone, magnesium sulfate, caffeine and caesarean.
