# Supplementary figures and images for: Third-day weight changes and bronchopulmonary dysplasia risk in preterm infants: a cohort study
Source: Front Pediatr. 2025 May 27;13:1592069. doi: 10.3389/fped.2025.1592069 (PMC12148873; doi:10.3389/fped.2025.1592069)

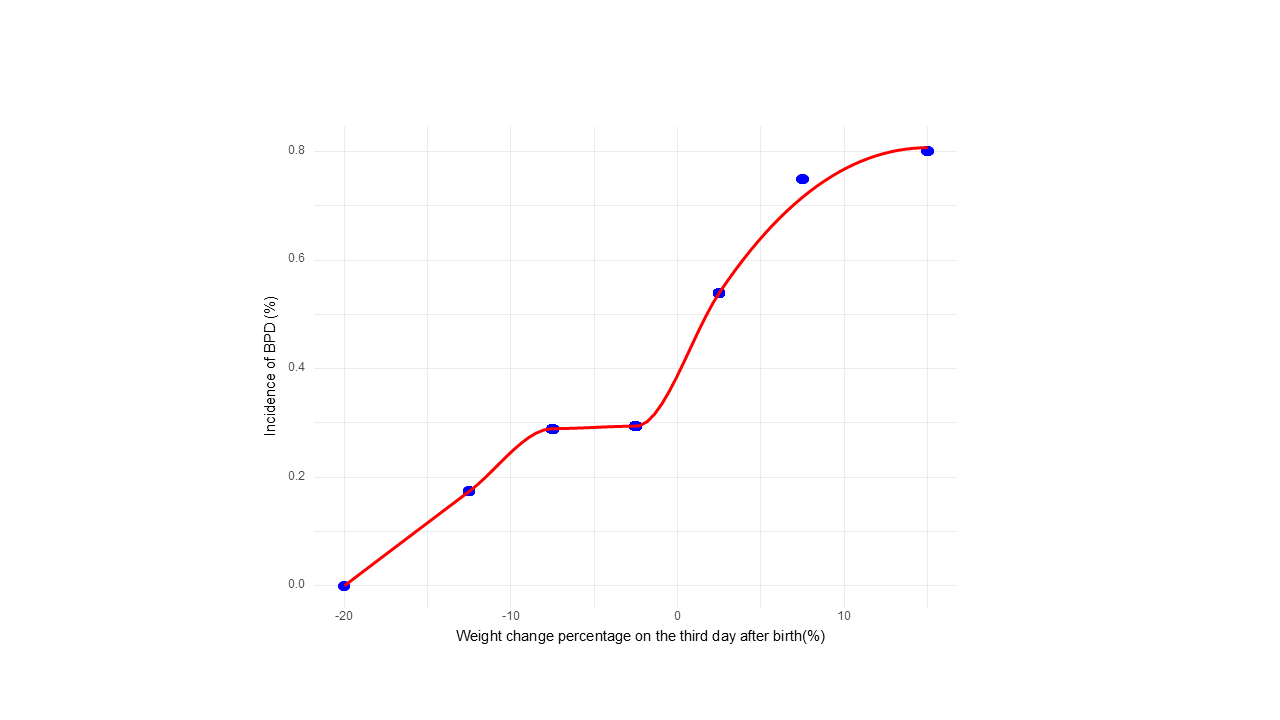

Supplement: Supplementary Figure S1 — Incidence of BPD by postnatal day 3 weight change categories. [file Image1.tif]
